# Landau model for illustrating the learning and unlearning process of nociplastic pain

**DOI:** 10.3389/fpain.2024.1307532

**Published:** 2024-02-20

**Authors:** Belén Valenzuela

**Affiliations:** Department of Theory and Simulation of Materials, Instituto de Ciencia de Materiales de Madrid, ICMM-CSIC, Madrid, Spain

**Keywords:** nociplastic pain, perception, biopsychosocial model, neurobiological education, Landau model, phase transition, mathematical psychology

## Abstract

Recent advancements in understanding the consolidation of nociplastic pain point to a complex, non-conscious learned process of threat perception. Neurobiological pain education is emerging as a promising approach to unlearn nociplastic pain, supported by biopsychosocial tools such as exposure to movement, mindfulness, and group sharing formats. However, this approach is still not well-known among clinicians and the society at large, creating a communication problem that unfortunately perpetuates the suffering of patients. Herein, we propose a Landau model to describe the learning and unlearning process of nociplastic pain, aiming to clarify this complex situation and facilitate communication across different sectors of the society. Nociplastic pain corresponds to a first-order transition, with attention more likely in the alert-protection state than in the trust-explore state. Two appealing results of the model are that the perception of the critical context depends on personal history regarding the symptom and that biopsychosocial loops are formed when there is alarming learned historical information about the symptom, along with confused and contradictory expert information, as seen in nocebo messages. Learning and unlearning in the model correspond to a chang in control parametrs that can weigh more on the alert-protection state, trust-explore state, uncertain state or neutral state. This description clarifies why neurobiological education is the foundational therapy from which others must be built to embody the accessible, clear, and trustworthy information.

## 1 Introduction

Chronic pain is increasing at an alarming rate in recent years, as exemplified by the prevalence of low back pain (1). Musculoskeletal chronic pain has been identified as a leading cause of disability worldwide (2). In addition, musculoskeletal conditions may elevate the risk of other chronic diseases such as cardiovascular disease, cancer, and diabetes (3). This disturbing situation has increased the research interest in gaining a deeper understanding of chronic pain. Recently, nociplastic pain has been defined as a significant component of chronic pain not linked to tisular damage (4). This term was considered necessary because most chronic pain is non-specific, with neural mechanisms playing a major role, and it does not correspond to an underlying pathology in peripheral tissues (5, 6). From advances in cognitive and phenomenological sciences, there is compelling evidence that the consolidation of nociplastic pain is a complex, non-conscious learned process of threat perception that gives rise to maladaptive loops. This process can be shaped by alarming interpretations of the context, misbeliefs, expectations, learned habits, as well as confusing or misguided information from clinicians or experts (7–14). This insight opens up the possibility to mitigate or alleviate nociplastic pain by potentially unlearning these beliefs and habits through the plasticity of the nervous system, thereby reducing the perception of threat. Biopsychosocial models rooted in neurobiological education (NBE) of pain (15–21) have emerged as the ground approach to face this situation.

Unlearning nociplastic pain through neurobiological education is not solely an intellectual process; it is an embodied one. This process involves establishing a safe and caring environment in which information is internalized from the conscious patient to the non-conscious organism until it becomes an automatic perception. Consequently, the patient makes sense of their own experience, understands how the organism works, and develops an internal compass to differentiate what constitutes a threat, what does not, and what remains uncertain. Reducing the perception of threat leads to a decrease in symptom intensity and frequency, improved functionality, and eventual symptom alleviation. However, this task is challenging as patients with nociplastic pain exhibit a non-conscious learned suffering pattern with intricate cognitive, emotional, attentional, motivational, motor, behavioral, and social loops (22).

Physiologically, the entire nervous system, including the brain, endocrine system, immune system, and even the microbiota, plays a role in perceiving threat (23, 24). Both innate and adaptive immune responses modulate pain perception and behavior (25). Therefore, the process of internalizing neurobiological education may vary for each patient and might take a different amount of time, necessitating a personalized approach (26). Considering the organism’s perception of threat, it is essential to establish a secure and supportive social environment for the patient, while also encouraging active patient engagement in their own recovery, which is vital for effective learning. This approach is often complemented with various techniques, tailored to the patient’s needs and preferences, to embody the information referred to as biopsychosocial tools (1). Examples of these tools include exposure to movement, mindfulness, group sharing formats, play, imaginative analgesia, psychological assistance, and, in general, exposure to a variety of activities (27).

Remarkably positive effects of this therapeutic approach have been observed in conditions such as migraine (28), musculoskeletal pain (26, 29–31), and fibromyalgia (32–35). This approach offers significant advantages, as patients are less exposed to the side effects often associated with prescription painkillers, which can induce addiction. Pharmacotherapy remains suboptimal (36), especially given the high placebo effects (12, 37). Ultimately, this embodied learning aids in understanding that hypervigilance, anxiety, depression, anger, fear, and catastrophizing in the pain experience are part of the process (38). Moreover, it sheds light on the possibility that other distressing symptoms experienced by the patient, such as insomnia, brain fog, ruminating thoughts, tense jaw, restless legs, tense muscles, digestive disorders, chronic fatigue syndrome, irritable bowel syndrome, and exteroceptionphobia, may also be linked to the perception of threat (16, 39).

Despite the numerous advantages offered by the biopsychosocial framework, its widespread integration remains limited within the healthcare system and the society at large. The reason is complex, and we will mention some aspects. First, pain is in the process of being understood, with its definition continually evolving (40–42). Pain is a complex subject addressed across multiple levels, encompassing biochemistry, physiology, psychology, sociology, and philosophy, with each level possessing its own intricacies and terminology. In addition, the vast volume, complexity, and generation of research information regarding pain make it challenging for essential insights to reach different sectors of the scientific community, clinicians, and, ultimately, the society. This situation gives rise to a communication problem, manifesting as a lack of dissemination of advancements in pain comprehension to both graduate studies (43) and the expert community. At the end of the chain, this uncertainty is translated to the patient, increasing their threat perception that fuels the pain. Furthermore, individuals suffering with chronic pain in an attempt to get rid of their diverse array of symptoms often seek help from various experts, whether clinicians or alternative specialists, increasing their already confused state. These experts and clinicians are referred to as the “expert culture,” a term borrowed from Arturo Goicoechea (44).

Given this scenario, it is not surprising that the neurobiological education proposal is poorly known. Unfortunately, this fact facilitates that patients absorb erroneous beliefs, many which are adopted from the expert community (7, 45–49). Common misconceptions translated to the patients are “pain is related to tisular damage,” or “the sensation of pain is proportional to tisular damage.” Another concern is fragility messages, such as “you have pain because your muscles are weak” (50). These misleading messages, often referred to as the nocebo effect, precipitate the consolidation of persistent pain (51). This is even more important due to the bias of the mind toward threat messages (52). The aforementioned uncertainties, coupled with these misconceptions, form a larger social loop where the patient is embedded. In these circumstances, it is important to recognize that pain is not exclusively determined by maladaptive loops within the patient. Instead, the patient’s loops have adapted to their misinformed social environment. This perspective aligns with newer definitions of health, emphasizing the organism’s adaptation to the accessible biopsychosocial information (42, 53). In this context, these biopsychosocial loops, characterized by unnecessary patterns of suffering, may adapt to societal influences but ultimately prove maladaptive to the potential wellbeing individuals can achieve through a deeper understanding of the workings of life.

Implementing the biopsychosocial model is also challenging: (1) Skepticism prevails within both the expert medical community and among patients concerning the proposition that pain can be acquired unconsciously and subsequently alleviated through the acquisition of knowledge in neurobiology of pain. Indeed, it is noteworthy that in-depth education in embodied neurobiology can yield significant benefits for an individual’s wellbeing. Consequently, achieving a consensus among experts regarding these concepts is paramount to engender trust and foster understanding. (2) Mere integration of a novel pain-focused curriculum falls short in effectively preparing medical students; it necessitates cultivating both competence and compassion toward their patients (54). Considering the intricate relationship between pain and threat perception, whether consciously or unconsciously perceived, establishing a reliable environment where patients experience being heard, believed, and comprehended forms the foundational step for initiating embodied learning of NBE. (3) The biopsychosocial model itself suffers from a vague definition, often resulting in the tendency to segregate the patient into three domains (biological, psychological, and social) without fully considering the patient’s subjective experience (50, 55). As highlighted by Stillwell and Harman (50), there is a tendency to utilize a reductionist approach when elucidating pain during patient education. Problematic pain explanations such as “pain is in the brain” may be employed, potentially causing confusion among patients who might think there is a problem within their cognitive capacities or undermining the reality of their pain (50). Instead, Stilwell’s proposition, based on the enactive approach, advocates for comprehending the subjective experience of the patient. The enactive perspective (56, 57) is a branch of embodied cognitive sciences based on dynamical systems, phenomenology, and organizational approaches to biology. It aims to build a bridge between life and mind, investigating organisms embedded in their physical and social context. In this approach, cognition is defined as “sense-making,” the capacity of an organism to evaluate different possible options and act in an adaptive manner to maintain and expand life.

In Granan’s study (58), it was proposed that approaches from the adjacent field of Statistical Physics, allowing the modeling of phase transitions, provide a suitable framework for comprehending chronification of pain and could be employed as a communication tool. The idea put forward was to build an Ising model to collect the positive and negative biopsychosocial factors relevant to pain, although a formal formulation of this model was not presented. We also think that the analogy to phase transition is useful in illustrating the essential understanding of chronification of pain; however, instead of focusing on positive and negative biopsychosocial factors from an external perspective, we propose to start from the subjective experience of the person. Remarkably, this perspective will also allow the illustration of unlearning the perception of threat in nociplastic pain. We favor the phenomenological Landau approach (59) to phase transitions as a starting point because it helps discern the essential variables and parameters. It is also simpler, what makes it more attractive as a communication tool in diverse disciplines and to different sectors of the society. In addition, it is possible to connect Landau models with Ising models, where Ising models serve as the microscopic version of Landau models (60).

In this article, we present a Landau model for automatic perception, which can manifest in an alert-protected state, a trust-explore state, or a neutral state. This is determined by the following parameters of embodied information: information from senses about the context, the historical experience of the patient related to the symptom, and the information from expert community that may influence and polarize the patient’s perspective. The equivalent of the free energy (59) is the patient’s sense-making. This is a term borrowed from the Enactive approach (61). In the model, automatic attention is located within the most likely state in the sense-making landscape. Several sense-making landscapes corresponding to different subjective experiences arise depending on the following parameters: Zen, uncertain, baby, hypervigilance, catastrophizing, curiosity, communicative, etc. As a result, the following are obtained: (1) The critical context from where the alert-protected or trust-explore states arise depends on the personal history related to the symptom. This result is consistent with recent knowledge in neuroscience (62, 63). (2) A hysteresis loop is formed with the personal history and contradictory or misguided expert information. This hysteresis loop corresponds to the biopsychosocial loops found in patients (19, 31, 44, 64). The model is used to illustrate the non-conscious learning process of nociplastic pain with nocebo messages and the embodied learning of neurobiological education to dissolve the biopsychosocial loop. The model might facilitate the communication of synthesized information through a unified framework and guide practitioners and health policies. In addition, it could enable the patient to make sense of their own experience. It can also serve as a tool to disseminate the benefits of a meaningful and updated biopsychosocial integrated framework to the society.

In the following section, we outline the derivation of the Landau model for automatic perception. Subsequently, we encompass various sense-making landscapes. We also show the creation of hysteresis loops, incorporating expert information and historical data. We illustrate the process of learning/unlearning nociplastic pain utilizing the model, and finally, we conclude with a discussion and summary.

## 2 Derivation of the Landau model of automatic perception

Landau models (59) were originally proposed to describe phenomenological phase transitions common in nature where a control parameter varies, for instance, the magnetization of iron as the temperature decreases below a critical temperature or when increasing a magnetic field. Magnetization would be the order parameter that is zero above the transition temperature and different from zero below the transition. The temperature and the magnetic field are control parameters that when varied can make a transition from one state to the other. Free energy is a functional of the order parameter and the control parameters whose minima determine the most stable states. The representation for a given set of parameters is a free-energy landscape with minimum points that correspond to the most likely states and will determine the state of the system. In the case of magnetization, there would be three possible states: downward magnetization, upward magnetization, and neutral state. An influential and inspiring article by Anderson (65) in the context of condensed matter physics proposed that the concept of phase transition could offer insights into understanding emergent phenomena from interacting components at each hierarchical level of science, including life and mind.

Most common phase transitions are of the first or second order in the Landau classification (66). In first-order transitions, there is a mixed state at the transition. For example, in the case of magnetization, there would be a mix between upward and downward magnetization. In second-order phase transition, there is, however, criticality at the transition; a very important concept that is related to large-scale cooperative phenomena. In the case of magnetization, at the critical transition, all the magnetic moments cooperatively align in either upward or downward magnetization and the magnetic susceptibility diverges. Extensions of the concept of criticality are widely used to describe life systems (67–69) and neural activity (70–72), where, for example, extensions of the Landau theory have been used to model cortex dynamics (73, 74). Psychodynamic processes have also a long tradition in dynamical systems (75–77), where, for instance, we can find that the Landau theory was used to model subjective experiences (78).

Now, we proceed to use the Landau framework to build up a model to delineate the process of learning/unlearning nociplastic pain. For that, we need to address the threat perception of the symptom. We will use inputs from phenomenology and cognitive sciences about the perception of pain or other symptoms related to an alert-protected state. It is not the scope of this work to achieve a comprehension of the complex process of perception of a sensation; instead, we just borrow some intuitive concepts from the scientific literature to present the phenomenological model.

Let us start by the sensation. We understand pain and symptoms as persistent sensations. These sensations are experienced throughout the day, reporting demands or needs from homeostasis and allostasis (53, 79). Thus, they represent a non-conscious evaluation of the organism’s needs. Physiologically, the information required for this evaluation is circulating through the neuro-immune-endocrine plus microbiota system. This includes cognitive–emotional information from an individual’s own history, context, and culture. All these give rise to a pattern of intricate rules aiming to ensure survival and expansion. The sensation is expressed in our consciousness and urges us to interact with the external world to satisfy the need as an automatic response. For instance, the sensation of hunger urges us to seek food. We perceive the sensation with the evaluation that the sensation might be hunger, and feel motivated to go for food. Consciously, we have the ability to decide whether or not to act upon this motivation. To describe a sensation, we need to consider both its valence and arousal (80, 81). Valence is associated with how the organism validates the sensation, whether it is pleasant (positive) or unpleasant (negative). Arousal measures the intensity of the sensation, if modulation is low, the sensation is felt quietly, and if it is high, it is felt agitated. Zero arousal corresponds to a neutral sensation.

Next, we address the automatic perception we want to model. The automatic perception of the symptom, denoted by ϕ, is chosen to be the order parameter of the Landau model. Among all the available information, it selectively collects the most relevant aspects to survival and expand. We will understand this automatic perception as a semiconscious cognitive–emotional evaluation of the symptom where the historical, sensorial, and expert information is integrated to discern the evaluation-motivational state: either the alert-protection or the trust-explore state (62, 79, 82). By semiconscious, we mean that it is possible to become aware of this semiconscious evaluation by self-observation. The perception of the symptom ϕ is equal to zero means the symptom is evaluated as neutral and there is no need for any action. If ϕ≠0, there is uncertainty either because there is a novelty or an inconsistency or a contradiction in the information perceived. Information serves to reduce uncertainty, prompting a cognitive–emotional causal inquiry within the default mode of the mind. This inquiry seeks to make sense either by recalling intrinsic information from one’s own history or by seeking extrinsic information related to the symptom from the context and social milieu. The evaluation can yield a negative value for ϕ, implying that the sensation is potentially threatening for survival and thus necessitates a protective response. Conversely, a positive value of ϕ corresponds to a perception of vitality, signifying a sense of safety and encouraging exploratory behavior.

Naively, one might expect that when the valence of a sensation is negative (an unpleasant sensation), then ϕ would be less than zero, especially if arousal is high. However, it is also plausible that the perception of an unpleasant sensation could transition to a neutral state over time. For instance, consider a scenario where a person has engaged in exercise and experiences stiff muscles. Internally, past experiences and knowledge about others’ encounters with stiff muscles after exercise are recalled. The automatic perception concludes that the sensation is familiar and will fade away. Consequently, no alarm is triggered for the individual, and minimal attention is devoted to the stiff muscles. At a certain point, ϕ might shift to a neutral state, eventually resulting in a decrease in arousal of the sensation.

Hence, there are several layers of evaluations: the non-conscious evaluation from the organism expressed in the consciousness by the sensation, the semiconscious automatic perception of the sensation ϕ, and the conscious agent perception. The conscious agent’s perception essentially involves a re-evaluation of the automatic perception and sensation within the current social and physical context. This allows for discernment regarding whether to follow the automatism or act in a different manner. These layers of evaluations might be confused or contradictory. For example, in nociplastic pain, when the agents wish to do their daily task, the organism evaluates pain and alert-protection perception and the agent cannot perform the task. In other words, it is not possible for the agent’s intention to translated into action, highlighting a misalignment between the agent and the organism. Alignment might be re-established through a conscious embodiment of information from neurobiological education. We will not model this feedback between the agent and the organism, just the automatic perception of the symptom from which the learning/unlearning process can be understood. The only conscious component that can be located in the model will be the conscious attention in contrast to the automatic attention.

Having defined the automatic perception as the order parameter, ϕ, we are ready to build up the Landau model. In Statistical Physics, F represents the free energy, and a minimum in the free energy corresponds to the most likely state of the phase space of a system. Specifically, states with lower energy correspond to higher probability. Analogously, a hill denotes an unstable state. The energy landscape will change shape at the transition. In the present case, the analogous quantity to energy is sense-making S, a term borrowed from the enactive approach (56, 57). As previously mentioned, perceiving a sensation triggers a quest for understanding by recalling intrinsic information (derived from one’s personal history) or seeking extrinsic information (from the physical or social context) associated with the symptom. What makes more sense to survive or to expand is what determines the more likely state of the perception ϕ among the possibilities. The minus sign is because the higher the sense-making (maxima in the landscape) corresponds to the higher probability. To make analogy to the Landau theory, we prefer to add a minus sign in such a way that minima corresponds to likely states −S=F. Having this in mind, we will call the different landscapes the sense-making landscapes. For that, we express F expanded in powers of the order parameter ϕ as follows:

(1) $$-S=F=-h_{ext}\phi +\frac{a}{2}\phi^{2}+\frac{h_{int}}{3}\phi^{3}+\frac{b}{4}\phi^{4}$$

In the expression (Eq. 1) all control parameters, $h_{ext}$, $h_{int}$, a, and b, are non-conscious embodied information of causal relations to infer perception of the symptom, i.e., meaningful information for survival or living concerning the symptom. $h_{ext}$ denotes an external bias provided by the information from the expert culture about the symptom. To model the current scenario of threat perception in nociplastic pain, as outlined in the Introduction section, $h_{ext}>0$ corresponds to clear, precise, and up-to-date neurobiological information about pain, while $h_{ext}<0$ corresponds to confusing and nocebo messages. As previously emphasized, expert advice holds significant relevance, as it provides the needed information to address uncertainty regarding a patient’s health. This expert advice can come from clinicians or from anyone whom the patient deems as an expert. Next, $h_{int}$ represents the historical information associated with the symptom, encompassing previously learned rules: beliefs, expectations from past experiences, and acquired habits relevant to the particular symptom. Positive/negative $h_{int}$ corresponds to alarming/pleasant information related to the symptom. Then, we define the parameter a following the common convention in the Landau theory, expressed as $a=a_{0}(T-T_{0})$. Here, T represents the registered information acquired through exteroceptive and proprioceptive senses, relating to the current context and the individual’s presence within this context. $T_{0}$ signifies the critical value indicating the threshold at which innate stored rules trigger a state of uncertainty within the context. A high value of T implies the collection of abundant information from the senses, while a low value of T signifies the collection of minimal information from the senses. When T is at zero, it denotes a complete absence of sensory information. Finally, both $a_{0}$ and b are innate positive parameters. By innate, we refer the genetic tendencies of the person. We will comment on these two last parameters in the Discussion section.

**Figure 1:**
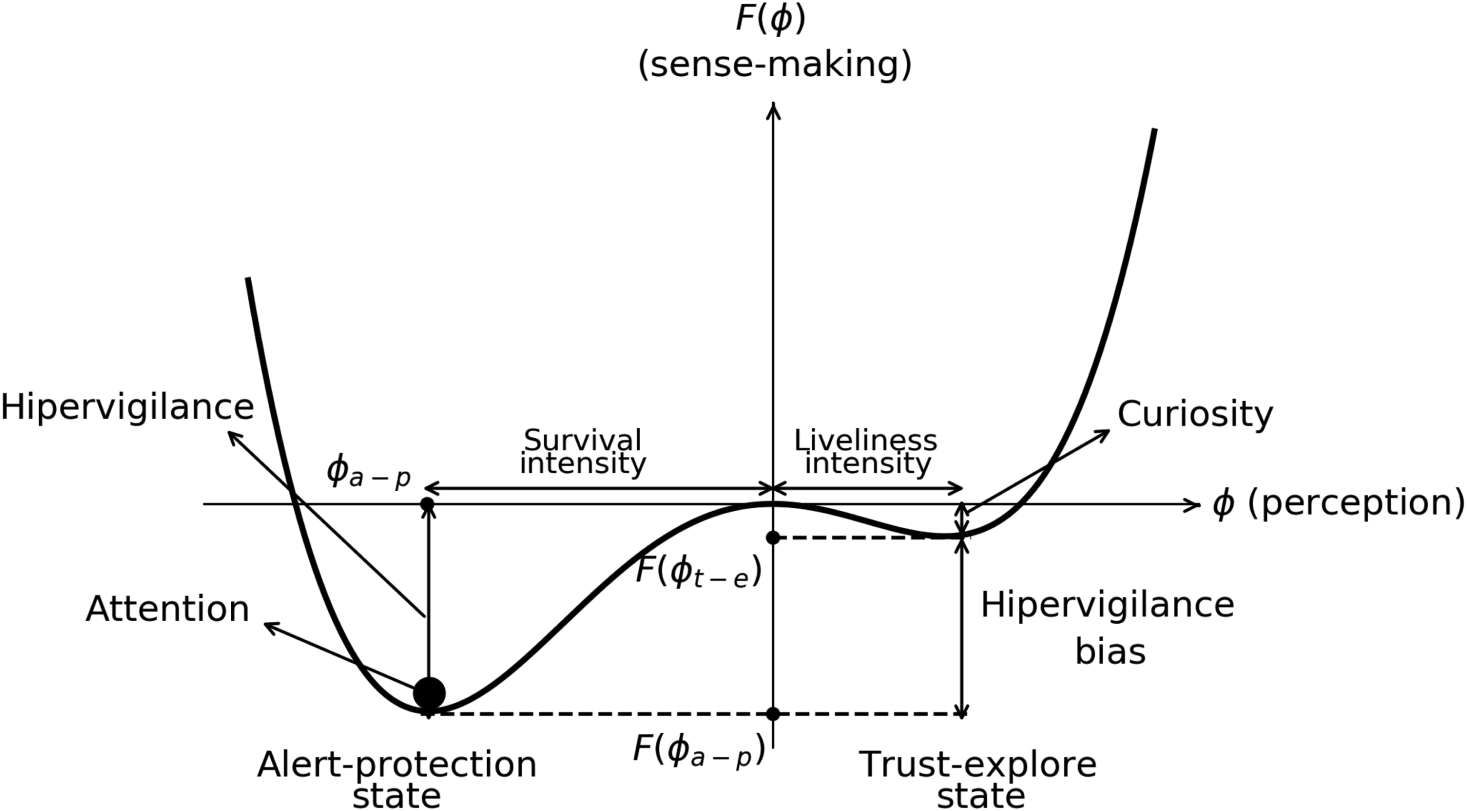
Typical sense-making landscape F(ϕ) vs the perception of the symptom ϕ showing deepest minima in the alert-protection state than in the trust-explore state. The black point is attention that is located in the deepest minima. Hypervigilance, curiosity, hypervigilance bias sense-making, and pleasant/unpleasant intensity in perception are depicted. Negative perception defines survival and positive perception liveliness.

In Figure 1, we represent a typical sense-making landscape F(ϕ) with two minima, enabling the definition of a useful vocabulary that encapsulates all the preceding concepts. Likewise, just as sensations possess valence and arousal, the perception of the sensation can also be categorized as positive, neutral, or negative. The magnitude of the perception corresponds to the absolute value of ϕ. A negative ϕ represents a perception associated with survival, while a positive ϕ corresponds to a perception associated with liveliness. The minima in the landscape signify the most probable perceptions. The minimum in the survival perception is termed the alert-protected state ($\phi_{a\text{-}p},F(\phi_{a\text{-}p}))$, and the minimum within the liveliness region is denoted as the trust-explore state ($\phi_{t\text{-}e},F(\phi_{t\text{-}e}))$. $\phi_{a\text{-}p}$ is the survival perception at the alert-protected state, and $\phi_{t\text{-}e}$ is the liveliness perception at the trust-explore state. The sense-making value at the alert-protected state is referred to as hypervigilance, $F(\phi_{a\text{-}p})$. Given the presence of an alert-protected state, what makes sense is to look for information regarding the potential danger. Conversely, the sense-making value at the trust-explore state is termed curiosity $F(\phi_{t\text{-}e})$. In an environment where trust is perceived, a natural curiosity arise to learn more about what is around the symptom. We also define two biases: the perception bias, $\Delta \phi =|\phi_{t\text{-}e}|-|\phi_{a\text{-}p}|$ defined as the difference between the intensity in the trust-explore state with respect to the alert-protection state, and the sense-making bias defined as the distance between the two minima, the trust-explore state respect to alert-protected state $\Delta F=|F(\phi_{t\text{-}e})|-|F(\phi_{a\text{-}p})|$. A positive bias in perception Δϕ>0 is optimistic, and a negative bias in perception Δϕ<0 is pessimistic. A positive bias in sense-making ΔF>0 represents curiosity bias, and a negative bias in sense-making ΔF<0 represents hypervigilance bias. Attention is depicted as a black point on the landscape. In the case of automatic attention, it is more likely to be in the global minimum. However, conscious attention can manifest at any extreme of the sense-making landscape, depending on the person’s will, although it might require more effort depending on the bias size. We finally define $\phi_{dm,a\text{-}p}(T=0)$ and $\phi_{dm,t\text{-}e}(T=0)$, not shown in the figure, that corresponds to a saturated perception where the mind is in complete default mode in either the alert-protection state or the trust-explore state. The saturated perception appears when there is no information from senses T=0 or there is some T≠0 but there is enough $h_{ext}$ such that $\phi_{dm,t\text{-}e}(T=0,h_{ext}=0)=\phi (T,h_{ext})$. This saturation perception will appear in the hysteresis loops representing biopsychosocial loops.

## 3 Sense-making landscapes

In the following, we analyze different sense-making landscapes available in the model depending on different possible perceptions, sense-makings, and biases. We identify the landscapes with mind bodysets in chronic pain such as hypervigilance and catastrophizing and with expansive states such as curiosity and communicative. Notice that different states also give rise to a particular social behavior that would be alert-protection–isolation and trust-explore–play.

**Figure 2:**
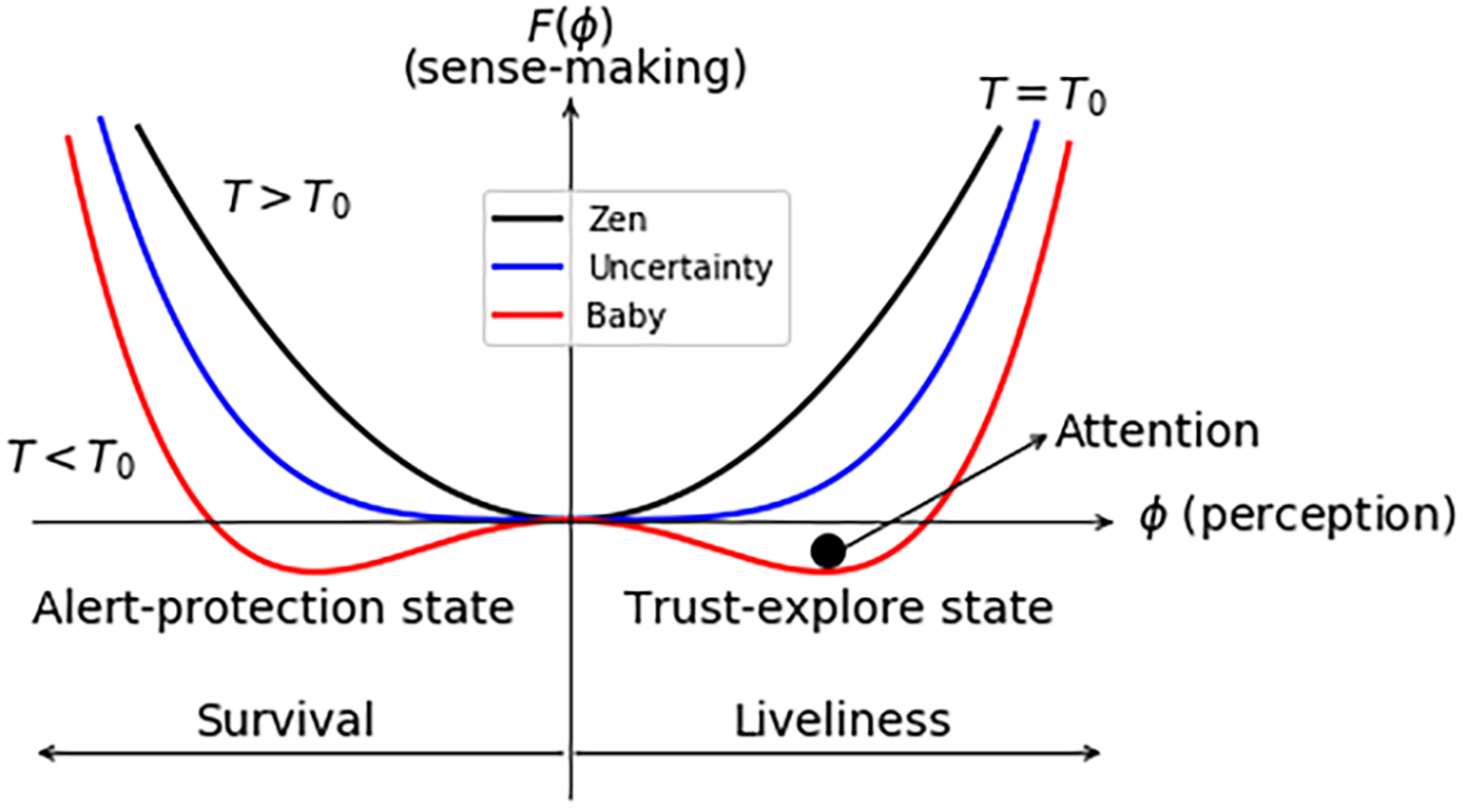
Sense-making landscapes for $h_{int}=0$ and $h_{ext}=0$. $T_{0}$ is the critical context. For $T>T_{0}$, the most likely possibility is the minimum at the neutral state corresponding to the Zen landscape. At $T=T_{0}$, information from senses is equal to the critical context and corresponds to uncertainty landscape. When the information from senses is below the critical context, $T<T_{0}$, alert-protection and trust-explore states have equal sense-making values. This is the baby landscape. Attention is depicted as the black point in the trust-explore state.

Let us first analyze the simplest case with no expert information, $h_{ext}=0$, and no historical information, $h_{int}=0$. Figure 2 shows this scenario with three different landscapes. This is the typical free energy of a second-order phase transition (66). Given the absence of prior information about the sensation, the perception aligns with the sensation from the organism. Therefore, what is felt in the sensation and perceived share the same valence and intensity. In this case, $T_{0}$ is the critical context from a neutral state to an uncertain state. When the information from senses is bigger than the one focused on the critical context, $T>T_{0}$, there is a minimum at the neutral state ϕ=0. This minimum is the only possibility for attention. The sensation is perceived as neutral. We will call this landscape the Zen landscape. Then, $T=T_{0}$ (blue line) is the critical value where uncertainty about the sensation sets in: the uncertainty landscape. At this value, there are as many minima on the left as there are on the right, which can be interpreted as having the same level of uncertainty in terms of threat or safety. Then, below the transition $T<T_{0}$ in Figure 1 (red line), there is less information from senses to focus perception on the sensation. The landscape corresponds to a balance between the alert-protected state (left-minimum) and the trust-explore state (right-minimum), and the attention has the same probability to be in any state. We have chosen attention, the black point in the landscape, to be on the trust-explore state. The state is balanced in the sense that the sense-making values at the minima are equal $F(\phi_{a\text{-}p})=F(\phi_{t\text{-}e})$. There is not perception bias, Δϕ=0, and no sense-making bias, ΔF=0. We will call this landscape the baby landscape. Thus, if the baby feels afraid, the attention is on the left minimum $\phi_{a\text{-}p}$, and if the baby feels safe and is willing to explore, the attention goes to the right minimum $\phi_{t\text{-}e}$.

**Figure 3:**
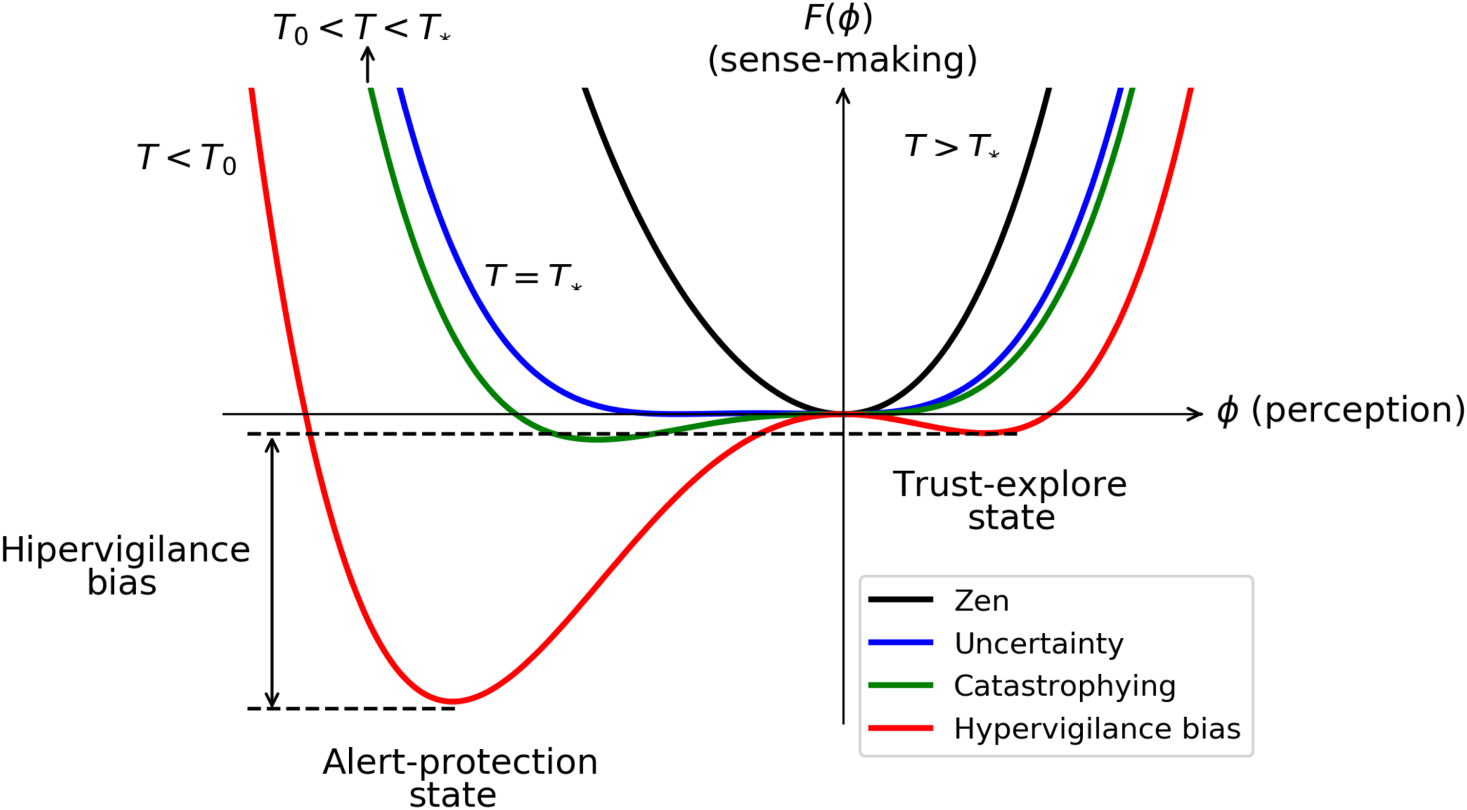
Sense-making landscapes for $h_{int}>0$. $T_{*}=T_{0}+\frac{2h_{int}^{2}}{9a_{0}b}$ is the new critical context showing how the perception of the context depends on alarming previous rules. For $T>T_{*}$, there is the Zen landscape, $T=T_{*}$ corresponds to uncertainty with survival bias, $T_{0}<T<T_{*}$ corresponds to the catastrophizing sense-making landscape, and $T<T_{0}$ corresponds to hypervigilance bias.

Next, let us consider the scenario where $h_{int}\neq 0$, signifying the presence of previously learned rules that influence the perception of the sensation. To illustrate nociplastic pain, we set $h_{int}>0$. We remind that positive $h_{int}$ comes from alarming non-conscious rules related to the sensation by the organism. The possible sense-making landscapes are represented in Figure 3. This landscape is the typical free energy of a first-order transition (66). In this case, $T_{0}$ does not correspond to the critical information from senses representing uncertainty, but $T_{*}=T_{0}+\frac{2h_{int}^{2}}{9a_{0}b}$. From this expression, it is seen that if there are many rules related to the symptom, i.e., $h_{int}$, big, this affect the critical context for uncertainty, i.e., $T_{*}$, big. This result agrees with studies in cognitive sciences (62, 63), where it is observed that alarming beliefs ($h_{int}>0$) distorts the perception of how danger is the context. We call this blue landscape in Figure 3 uncertainty pessimistic bias with multiple minima at F=0, more abundant on the left than on the right. This configuration implies that attention can fluctuate and shift between these multiple minima. At $T>T_{*}$ (black line), we just have a minimum, and this state corresponds to the Zen landscape, as we have explained above. Attention can just be in the neutral state. For $T_{0}<T<T_{*}$, the organism is just in an alert-protected state that we have assigned it to the catastrophizing landscape. Attention is in the alert-protected state. Here, there is pessimistic bias Δϕ<0, hypervigilance bias ΔF<0, and no curiosity $F(\phi_{t\text{-}e})=0$. At $T<T_{0}$ (red line), there is a mixed state again with a pessimistic bias Δϕ<0 and an hypervigilance bias ΔF<0 but with some curiosity in such a way that attention is more likely to be in the alert-protection state than in the trust-explore state. In this example, hypervigilance bias means that there is a tendency to absorb alarmed messages about the symptom. Notice that to focus on just information related to the symptom means lower information from senses (T is lower). Therefore, this mixed state is called the hypervigilance bias landscape.

Let us consider now the case with $h_{ext}\neq 0$. If $h_{int}=0$, the image represented in Figure 2 (red line) will have lower minima in the alert-protection or the trust-explore state depending on the sign of $h_{ext}$. If this case represents a baby, $h_{ext}$ would typically represent the parents that polarize the baby uncertainty. If $h_{int}\neq 0$ and focuses in illustrating the case of nociplastic pain, $h_{ext}$ is the information from expert culture with strong impact in reducing uncertainty. In this case, $h_{ext}$ can polarize the perception of the patient. We remind that $h_{ext}<0$ denotes misinformed information by the expert culture, while $h_{ext}>0$ corresponds to updated expert information in relation to the integrity of tissues and knowledge of pain mechanisms. Of course, in a general case, $h_{ext}>0$ might be also misinformed information representing placebo effect, but we stick to the first situation to describe the nocebo problem in nociplastic pain. A negative value of $h_{ext}$ favors the alert-protected state, as in the case of $h_{int}>0$ shown in Figure 3. The explanation of different states would be similar where, in addition to historical alarming beliefs and maladaptive habits, there are misinformed messages from expert culture and proposition of rigid habits.

**Figure 4:**
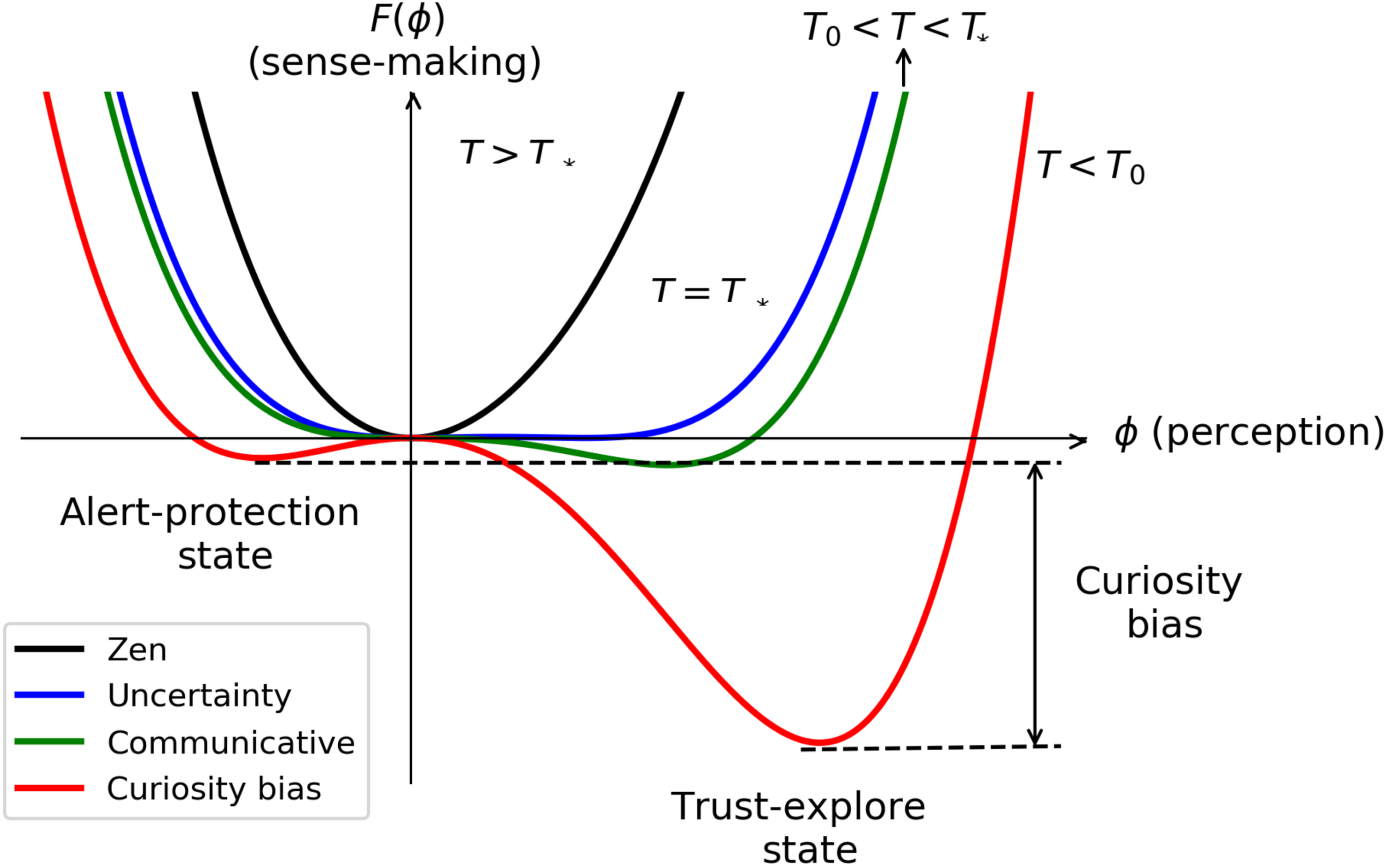
Sense-making landscapes for $h_{int}<0$. The critical context $T^{*}$ depends now on comforting previous rules in $h_{int}$. Zen landscape for $T>T_{*}$, $T=T_{*}$ corresponds to the uncertainty with liveliness bias, $T_{0}<T<T_{*}$ corresponds to the communicative sense-making landscape, and $T<T_{0}$ corresponds to curiosity bias.

In Figure 4, we illustrate the scenario where $h_{ext}>0$, corresponding to updated expert information, and/or $h_{int}<0$, corresponding to reliable, safe, and comforting learned rules about trust in the organism. Again $T_{0}$ becomes $T_{*}=T_{0}+\frac{2h_{int}^{2}}{9a_{0}b}$, meaning that there is an optimistic bias to perceive the surround at the critical context. In this case, the landscape at $T_{0}<T<T_{*}$ represents the communicative sense-making landscape where the person is willing to share their discoveries about how to recover from the symptoms. In this situation, we observe an optimistic bias with Δϕ>0, a curiosity bias with ΔF>0, and no probability for threat indicated by $F(\phi_{a\text{-}p})=0$. $T<T_{0}$ (red line) corresponds to a mixed state, but now the global minimum is in the trust-explore state and there are again both optimistic bias Δϕ>0 and curiosity bias ΔF>0, with attention more likely in the trust-explore state than in the alert-protection state.

In summary, when both $h_{int}$ and $h_{ext}$ are zero, we observe a second-order phase transition, resulting in the formation of three landscapes as the information from the senses decreases: Zen, uncertainty at the critical context $T_{0}$, and baby landscapes. In this scenario, the sensations and the automatic perception of the individual share the same valence and intensity, and there is no bias. When $h_{int}$ is different from zero, there are first-order transitions. The critical information from senses to arrive to an uncertain state is $T_{*}=T_{0}+\frac{2h_{int}^{2}}{9a_{0}b}$, meaning that the critical context depends on the historical rules with respect to the symptom $h_{int}$. In this scenario, mixed states emerge with biases in both perception and sense-making. If the perception bias leans toward pessimism, as information from the senses decreases (T decreasing), the landscapes encountered first feature a catastrophizing landscape with zero probability for the trust-explore state, followed by a hypervigilance bias landscape, emphasizing the importance of focusing on information for self-protection. Conversely, if the perception bias is optimistic, the progression with decreasing sensory information involves a communicative landscape with zero probability for the alert-protected state, followed by a curiosity bias landscape, encouraging exploration and curiosity.

Which landscape is the most appropriated? The evaluation of the automatic perception is done with the information circulating within the organism (53). If the information is misleading, there might be a wrong evaluation. Opportunities of potential wellbeing thus need to be investigated. In the following we will delve deep into an error in evaluation due to confused or erroneous messages from expert culture.

## 4 Hysteresis loop from expert information as a biopsychosocial loop

Let us imagine different criteria from the expert culture given to the patient, i.e., $h_{ext}$ varies for a given history of the person $h_{int}$ related to the symptom and context T. These scenarios characterized by first-order transitions resulting in mixed states correspond to the hypervigilance bias and the curiosity bias sense-making landscapes. We will see that hysteresis loops are formed in terms of perception ϕ concerning the information absorbed from the expert culture ($h_{ext}$). Within the hypervigilance loop, there exists a bias toward pessimistic information, whereas the curiosity loop exhibits a bias toward optimistic information. In the context of nociplastic pain, we have established a relationship where negative $h_{ext}$ is associated with the nocebo effect and positive $h_{ext}$ is linked with neurobiological education.

Mathematically, the hysteresis loops are obtained from the minimum of F with respect to perception to find the most likely states.

(2) $$\frac{\partial F}{\partial \phi }=0\to h_{ext}=a\phi +h_{int}\phi^{2}+b\phi^{3}$$

**Figure 5:**
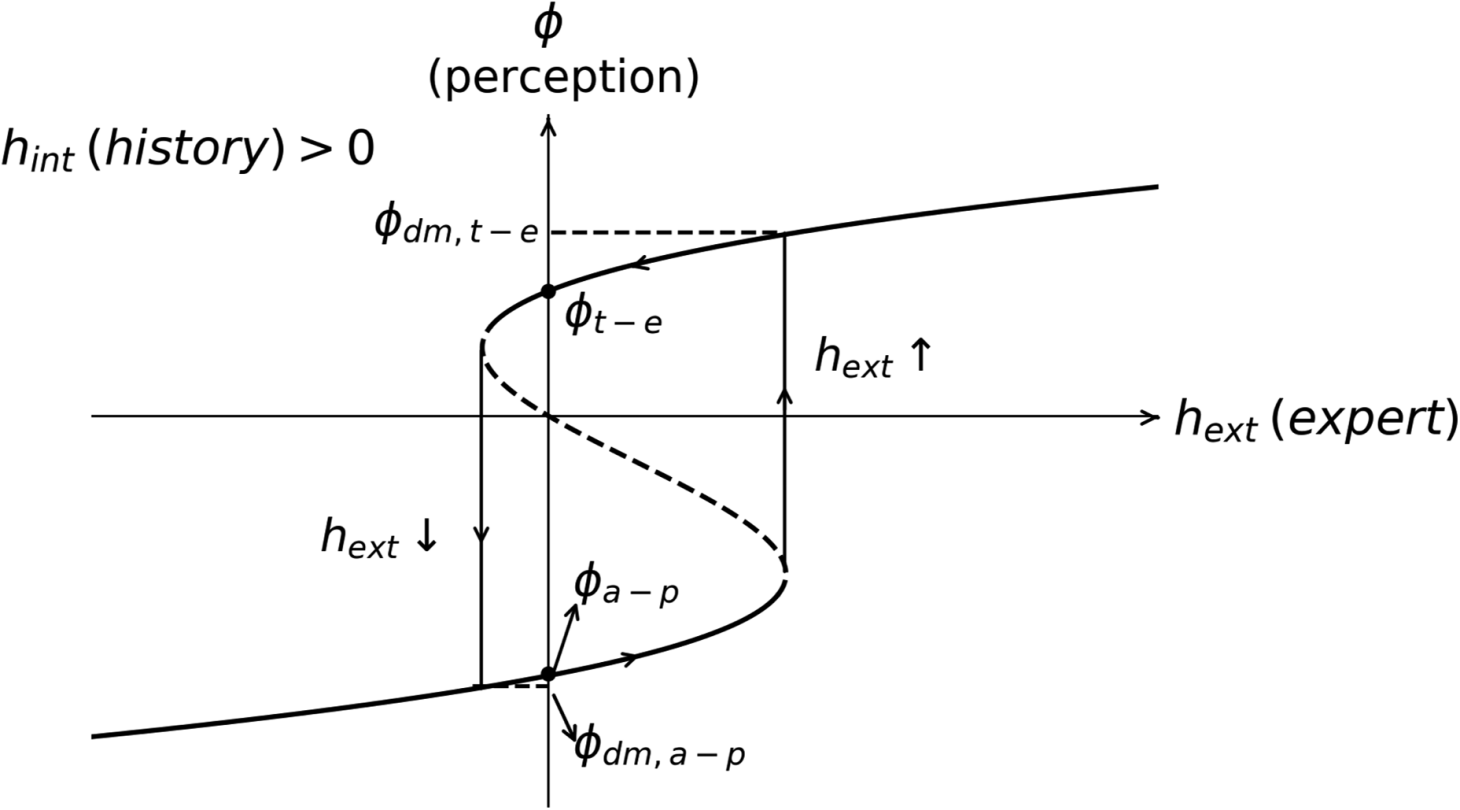
Hysteresis loop between the perception of the symptom and the expert embodied information $h_{ext}$ related to the symptom. Dashed lines do not belong to the loop because they correspond to the case where the polarization of the perception is opposite to the expert information. From the figure, it is observed that the loop is mostly in the alert-protection state since $h_{int}>0$ and the embodied neurobiological education $h_{ext}>0$ must have a big value to counteract the previous bias $h_{int}$. The default modes in the alert-protection state $\phi_{dm,a\text{-}p}$ and in trust-explore state $\phi_{dm,t\text{-}e}$ are depicted as well as the most likely perceptions $\phi_{dm,a\text{-}p}$, $\phi_{dm,t\text{-}e}$ at $h_{ext}=0$. $h_{ext↑}$/$h_{ext↓}$ represents the value of the embodied expert information when there is a transition from survival–liveliness/liveliness–survival perception.

The hysteresis loop from Equation 2 ϕ vs $h_{ext}$ in hypervigilance bias is represented in Figure 5. In the figure, the survival perception at the alert-protected state $\phi_{a\text{-}p}(h_{ext}=0)$, the liveliness perception at the trust-explore state $\phi_{t\text{-}e}(h=0)$, and the saturated perception corresponding to the default modes at the alert-protected state $\phi_{dm,a\text{-}p}$ and trust-explore state $\phi_{dm,t\text{-}e}$ are displayed. $h_{ext↑}$ is the absorbed expert information needed to go from the alert-protection state to the trust-explore state, and $h_{ext}↓$ is the absorbed expert information to change from the trust-explore state to the alert-protection state. In the case of nociplastic pain, $h_{ext}↓$ will correspond to the absorbed nocebo messages needed to change states from trust-explore to alert-protection and $h_{ext↑}$ to the absorbed neurobiological education needed to change from alert-protection to trust-explore.

The loop starts at $\phi_{t\text{-}e}(h=0)$ (see Figure 5). For negative $h_{ext}$, there are alarming messages and ϕ is decreasing toward $h_{ext}↓$. The dashed line has a negative slope, meaning that the trust/threat perception is opposed to information given by the expert culture $h_{ext}$ and then do not polarize the opinion of the patient: on the contrary, patient’s opinion is opposite to expert information. Since we have started with the assumption that the expert information polarizes the patient’s opinion, we do not consider this case. Then, at $h_{ext}↓$, the perception turns from trust-explore to the default mode of alert-protection $\phi_{dm,a\text{-}p}$. If neurobiological education is absorbed now, the threat perception is decreased until it finally reaches $h_{ext↑}$, where there is a change in the state to $\phi_{dm,t\text{-}e}$. Then, nocebo messages might be reminded or collected and the loop starts again.

The meaning of this hysteresis loop is that the patient is confused by the contradictory information between nocebo and neurobiological education. We associate the hysteresis loop with a biopsychosocial loop, where bio is from the symptom, psycho from perception, and social from information from expert culture $h_{ext}$. This loop gives rise to the cognitive, emotional, attentional, motivational, motor, and conductual loops that help consolidate the persistent symptom. This underscores the critical need for coherence in information across the expert community, media, universities, and schools to mitigate such confusion and its subsequent effects.

## 5 Learning and unlearning persistent pain illustrated by the model

In the model, unlearning nociplastic pain is just changing parameters: learning nocebo messages $h_{ext}$ to trustful and updated neurobiological information $h_{ext}^{\prime }$, alarming past learnt rules $h_{int}$ to new rules from revising meaning of previous ones, $h_{int}^{\prime }$, training senses to collect more sensorial information $T^{\prime }$, or training conscious attention to realize the no permanent evaluation of the perception states. Clearly, the patient is the main character taking an active coping in all the processes to embody the new information. Let us illustrate this process of learning/unlearning chronic pain with an hypothetical example.

Let us consider there is a perception ϕ of a sensation that it is an ache in the neck. If the neckache remains and there is uncertainty because the pain is new or disturbing, the organism goes from a neutral state to an uncertainty landscape $T=T^{*}$, blue line in Figure 3. The default mode of the mind will be wandering with ruminating thoughts correlating causal possibilities about the symptom. For example, do I need to worry about the neckache? I have been told that screens force a bad neck posture. Should I go to the doctor? Should I buy another screen/mouse? (attention is in the alert-protection state). Let us move a little bit or go for a walk (attention is in the trust-explore state). I think it is nothing to be worry about (attention is in the neutral state). There might be the possibility that there is a bad memory about neckache because there was some accident some years ago. In this case, $h_{int}>0$ and $T^{*}=T_{0}+(2h_{int}{)}^{2}/(9a_{0}b)$. Thus, the critical context is uncertainty with a pessimistic bias in perception. The interest about the neckache increases, and the patient, conscious or not, focuses on information about it, paying less attention to senses information, i.e., T decreases and the hypervigilance sense-making landscape is formed (red line in Figure 3). In principle, health experts have privilege information about pain, and when the patients goes to the health expert, they expect to make sense of their pain and especially to get relief from the symptoms. Consider a scenario where experts infer from an X-ray a cervical deviation and tell the patient that pain arises because the patient adopts a bad posture while working with computers. Current neurobiological knowledge highlights that there is neither a beneficial nor a harmful posture for pain (83, 84). The attribution between posture and pain and the rigid recommendation of a correct position can constitute a nocebo message, i.e., $h_{ext}<0$. A new fear about no correct position is leaking in the organism. The alert-protected state becomes deeper in the hypervigilance landscape shown in Figure 3 (red line). Pain might consolidate, becoming persistent and sensitive to sitting on a chair, i.e., context information included in $T^{*}$. This experience disrupts the person’s life since the organism finds danger at their workplace and other symptoms such as brain fog and intrusive thoughts might appear when trying to concentrate at work, giving rise to frustration. This will fuel the evaluation of threat, and other symptoms corresponding to the alert-protection state might arise such as tense jaw, insomnia, and digestive disorders. Each symptom will have its own sense-making landscape. The patient goes from one expert to another, but no tisular damage is found. At this point, the person is suffering and might distrust the expert community and distrust their organism (85).

Imagine now the person decides to visit a neurobiological education clinic. The information provided by the clinicians is different $h_{ext}^{\prime }>0$. At the beginning, there will be the biopsychosocial loop due to the contradictory information shown in Figure 5. How can the person trust NBE if the most likely state is the alert-protected state without trust in neither the expert community nor in their own organism? Moreover, if there is the additional factor of losing employment status because the patient is losing functionalities, the threat intensifies (13). Therefore, the first challenge is to build and maintain trust. The time needed to build trust will depend on the information embodied from own history $h_{int}$, from the experts $h_{ext}$, and from the context T. That is why, to build a safe and caring environment and a clear, accessible, and honest information about pain is so relevant. Then, when trust is built between the patient and the clinician, it becomes possible for an active coping of the patient and a compromise to go through the practice. Certainly, this trust building is necessary to start with but also during all the processes of unlearning the threat perception while embodying learning NBE.

Concurrently, the patient guided by clinicians explores that threat perception is not permanent playing with conscious attention, locating their present sense-making landscapes and inferring non-conscious rules in $h_{int}$. How non-conscious rules can be identified if precisely they are not conscious? When learning about NBE, there might be a contradiction between the new information and the person’s misbeliefs. The contradiction might be disturbing and leads to the uncertainty state and the biopsychosocial loop. These contradictions can be also identified when listening to the narrative of the patient, observing their body language and behavior. It is necessary to approach these contradictions gently and with empathy since, if not, alert-protection will emerge. Empathy might arise when the expert community realizes their own personal biopsychosocial loops maladaptive to life and understand how difficult it is to dissolve them. The patient can also become aware, via observing and exploring with curiosity instead of hypervigilance, the default mode of the mind and the own maladaptive cognitive, emotional, attentional, motivational, motor, and conductual loops. From this exploration, it might be also possible to infer misbelieves and maladaptive habits in $h_{int}$. In the present example, the patient will learn in NBE that many people with strong cervical deviation do not have any pain (correlation not equal to causality), will learn that there is no correct position but a position for each occasion, and will learn that movement is better than complete rest. The patient will also learn that all the symptoms stem from the threat perception, pinpointing the root of the problem (19, 31, 64). All this new learning contrasts with previous expert information. The updated information needs to be embodied and explored with curiosity, for example, by playing when the patient feels safe with any possible biopsychosocial tool available. Playing safely will also change how much information is extracted from the context, $T^{\prime }>T$. Notice that playing might encounter some resistance because what the person wants is to get rid of the pain. It is needed to remind that to embody the new information, it is necessary to explore without any objective, like a baby. Training senses increases T, which is beneficial for any misleading evaluation (see Figure 3). Furthermore, by training conscious attention, the patient becomes more aware of their own sense-making landscape, as it reveals that there are more states available than just the deepest minimum corresponding to automatic attention. A challenging issue is that in the process the person might arrive to the catastrophizing state where the patient feels that there is no hope. However, notice that the catastrophizing landscape is closer to the neutral state than the hypervigilance bias landscape (see Figure 3). We interpret this phenomenon as the “Phoenix effect,” wherein from the depths of overall suffering, a new perception emerges when information from the senses is allowed. Becoming aware of this state might be part of the process. In addition, resistance, pain, and symptoms may reappear from time to time, and all the unlearning process has to be initiated again but with a foundation of prior learning and experience. Patience is crucial, coupled with trust in one’s own organism. If finally the misbelief is dissolved, $h_{int}\to h_{int}^{\prime }$, the sense-making landscape will change accordingly going toward more probability in the trust-explore state than before and less probability in the alert-protection state (red line in Figure 4). Eventually, the sense-making landscape might be also communicative, where the patient is willing to tell their recovering experience (green line in Figure 4). The clinician might become aware by a different narrative and a different body language.

It is evident that the patient takes on the central role in their own recovery, guided by experts in NBE and supplementing with biopsychosocial tools tailored to the patient. The duration of the recovery process will vary for each individual (26). While there may be fluctuations in pain and occasional setbacks, the patient improves their functionality, subsequently enhancing their overall quality of life.

## 6 Discussion

The present Landau model describes phenomenologically and qualitatively key aspects in the perception of a symptom ϕ: (1) The contribution of personal history, physical context, and expert culture as control parameters to build the perception as order parameter. (2) Optimization of the sense-making to discern if perception should be in an alert-protected state, in a trust-explore state, or in a neutral state. (3) The automatic attention located in the deepest minima of the sense-making landscape and the conscious attention that could be located in any extrema of the sense-making landscape. (4) Second-order transitions are derived if there are not past learnt rules, $h_{int}=0$, and first-order transitions if there are past learned rules, $h_{int}\neq 0$. (5) There are various possible sense-making landscapes, allowing for the characterization of different stages of the subjective experience. For second-order transitions, these sense-making landscapes are Zen, uncertainty, and baby, and for first-order transitions, they are uncertainty bias, hypervigilance bias, catastrophizing, curiosity bias, and communicative. (6) Unlearning corresponds to a change of the parameters from nocebo messages to NBE education $h_{ext}\to h_{ext}^{\prime }$, changing the meaning of learned rules $h_{int}\to h_{int}^{\prime }$ and training senses $T\to T^{\prime }$. This process will allow us to change the perception.

As a result, in first-order transitions, the critical context $T_{*}=T_{0}+(2h_{int}{)}^{2}/(9a_{0}b)$ depends on the individual’s personal history concerning the symptom $h_{int}$. This aligns with neuroscience studies, which indicate that personal context is inferred based on an individual’s beliefs (62, 63). Another interesting finding is the formation of hysteresis loops when expert information is incorporated alongside historical information. This phenomenon is interpreted as the biopsychosocial loop, and it is in line with the previous proposal that hysteresis loops can help clarify the understanding of perception within the framework of neural representations (86). Interestingly, from a different perspective, there have been proposals using neural networks to explain some mental illness as a disruptions of criticality (70, 71), which agrees with the view of pathology as a first-order transition in this simplified model.

The critical context $T_{*}=T_{0}+(2h_{int}{)}^{2}/(9a_{0}b)$ also depends on the innate parameters $a_{0}$ and b. In Statistical Physics, $a_{0}$ is related to the susceptibility to the magnetic field $\chi ∼1/a_{0}$, and in the present model, would be the perception susceptibility to expert information. Thus, for a bigger $a_{0}$, there will be lower susceptibility to expert information and the extra term in the critical context $T^{*}$ will decrease, as might be expected. There might be people more sensitive to expert information (small $a_{0}$) than others (big $a_{0}$). However, b is related to self-interaction (60), self-perception in the present case. In the model, b cannot be very large because this would mean that other powers in perceptions would be necessary such as $\phi^{6}$. For a deep understanding of the consequences of these parameters and a precise cognitive definition of b, a thorough study connecting the Landau model with Statistical Physics is necessary (60). This will be left for future studies.

A clear limitation of the model lies in its static nature within Landau formalism. We introduce an effective dynamic by changing the control parameters reporting information from context, patient history, and expert culture concerning the symptom. This dynamic does not correspond to time dynamics since at each time there might be different sensations. A persistent sensation is just more likely in time. The dynamic corresponds to a variation in the embodied information $\delta h_{int}$, $\delta h_{ext}$, δT, which will be reflected in a variation of the perception. Another limitation is that the model does not include the negative/positive feedback loop that will arise in the hypervigilance/curiosity bias landscape. A non-equilibrium model will be necessary to address this effect. This study will also properly account for the probability of the trust-explore or alert-protection metastable states in the mixed state (66), which again requires the development of the model from first principles in Statistical Physics (60). However, the model provides a valuable coherent framework for the intended purpose of illustrating and communicating the learning and unlearning process of nociplastic pain.

The model is simple and intuitive, and it is phenomenological in the sense that it pretends to describe the common aspects about the experience of the perception of learning/unlearning nociplastic pain as sense-making landscapes. The model does not try to explain the perception of pain as, for example, the computational approach of the Bayesian brain (9). The Bayesian brain model perceives perception as primarily non-conscious prediction, modulated cognitively and built upon integrating sensory inputs, prior experiences, and contextual cues. This view aligns with the alert-protected state of the perception in our model and with the result commented above about the critical context. However, the Bayesian brain model can be likened to a “crooked” scientist, constantly seeking trajectories that minimize uncertainty (87). The Bayesian hypothesis inherently leans toward uncertainty reduction as the primary approach to dealing with uncertainty, leaving little room to explore other possibilities. On the contrary, curiosity embraces uncertainty and actively engages with it. This distinction is fundamental, as the Bayesian model lacks a direct analog to the trust-explore state, which is pivotal for unlearning pain.

The model clarifies why embodied neurobiological education goes to the core of the problem instead of just improving symptoms. Neurobiological education helps point out nocebo messages and other misconceptions and makes sense of patient’s experience. There are other approaches that aim to get rid of the symptoms, but pain reappears since misconceptions and the hipervigilance bias evaluation remains. That is, improving symptoms relieve the patient but do not change the landscapes; just attention shifts from the alert-protected state to the metastable trust-explore state or to the neutral state. Becoming aware of misconceptions and embodying the information with appropriated biopsychosocial tools do change the landscape. It also helps show how the patient actively copes with their condition by making sense of their experience and becoming aware of potential sense-making landscapes. The model also emphasizes the importance of establishing a shared knowledge base on pain among all clinicians, highlighting the need for a unified understanding among healthcare professionals to prevent biopsychosocial loops. Ultimately, the model illustrates the considerable utility of neurobiological education in preventing persistent pain and other related symptoms.

Extensions of the model can be used to address learning/unlearning of other mental syndromes such as anxiety, depression, functional or somatoform symptoms, and addictions, which seem to have a common underlying mechanism (79, 88). It is interesting to notice that biopsychosocial loops are in both, hypervigilance bias and curiosity bias, landscapes, as might happen with screen addictions where curiosity bias is looking for the sensation of surprise. This is in accordance with findings in cognitive science, where a balance between exploration and exploitation is considered adaptive, while over-exploration or over-exploitation is being indicative of maladaptive responding (see Krypotos et al. (89) and references therein). Exploitation is related to pursue what is already known and related to hypervigilance in our work. In curiosity bias, the patient instead of avoiding the sensation as in pain (hypervigilance bias) is looking for the sensation. The model could be also adapted to pathologies where the rules learnt by the organism opposes to the expert rules as, for example, in a maniac state. Anosognosia is common in mental syndromes, and it is not that surprising that if it would be possible for the patient to become aware of misbeliefs and mishabits, this might be of extreme relevance for the recovering of the patient.

The proposed model aligns with the allostasis perspective that mental syndromes are not simply the result of dysregulated processes; rather, this dysregulation frequently stems from the organism's embodied learned rules. Allostasis is defined then as stability through change to adapt to different needs of the organism (53). This requires prediction of the needs to satisfy them before they arise. Health is then defined as the capacity for adaptive variation, and disease is defined as a compression of this capacity, in contrast to the traditional definition of health as a list of “appropriate” lab values and disease as “inappropriate” values based on the control of homeostasis. The term allostatic load is used to refer to disease as a maladaptive loop behavior by the organism, which is not dysregulated but coherent with their own innate and learned rules. Allostasis thus enlarge the scope of health allowing to deal with cognitive and emotional symptoms. In this context, chronic pain has been described in terms of allostatic load (24, 90).

In long-term processes, however, the allostasis perspective of “stability through change” might not be enough since in the historical process of life there is no stability but a continuous transformation where a process of individuation might emerge. This is in line to the proposal of extending criticality and symmetry breaking, where the living state of matter is interpreted as an ongoing extended or critical transition, always transient to a renewed organism (69). We conceive the learning process in the line to the proposal given in the Enactive plus Simondonian approach (88), which emphasizes that “growth and transformation processes can arguably be seen as fundamental for self-individuation for humans, not only subsistence.” This devenir seems to be in line with the process of individuation proposed by Simondon as the generation of metastable states by transforming tensions in the environment or in the society (91).

In conclusion, we have built a Landau model to address the subjective perception of a patient, which can be in the neutral, alert-protected, or trust-explore states. The order parameter is the perception of a symptom, and the control parameters are the context from senses, the embodied history, and the embodied information from expert culture about the symptom. The model allows one to show different perception scenarios corresponding to different sense-making landscapes where automatic attention is placed in the most likely state. For second-order transitions, there are the Zen, uncertainty, and baby landscapes. First-order transitions present bias either for the alert-protected state or the trust-explore state, giving rise to other possible landscapes: uncertainty bias, hypervigilance bias, catastrophizing, curiosity bias, and communicative. From the model, two interesting results well-known in cognitive science are derived : (1) the critical context where uncertainty appears depends on non-conscious historical misconceptions and mishabits about the symptom and (2) an hysteresis loop named the biopsychosocial loop arises in perception when there is confused expert information together with non-conscious alarming historical information. We apply this model to illustrate the threat perception given in nociplastic pain and the unlearning process via embodied neurobiological education. Learning and unlearning correspond to changing control parameters, namely, a revision of non-conscious misconceptions and mishabits, updating with trustful expert information, and training senses and attention.

From this model, it is clearly seen that the alarming increasing rate of chronic pain could be partly explained by nocebo and confused expert information that creates a threat perception in the patient and precipitates the organism into an alert-protection state. Within the embodied learning of NBE, the patient might identify these nocebo messages, investigate their own sense-making landscape, and infer their own alarming beliefs and mishabits. Embodied learning of neurobiological education emerges as a valuable tool to reduce the perception of threat, prevent the chronic pain burden, and antifragilize citizens who develop their own internal compass to be in the world. The strongest policy effort will be to promote this embodied neurobiological education, besides clinicians, to the whole society from schools to universities and media. This will avoid loops from the nocebo effect, value the importance of the trust-explore state, and encourage individuals to make sense of their own experience.

## Data Availability

The original contributions presented in the study are included in the article/supplementary material, further inquiries can be directed to the corresponding author.
